# Allelic imbalance in human breast cancer

**DOI:** 10.18632/oncotarget.14648

**Published:** 2017-01-13

**Authors:** Juliet D. French, Stacey L. Edwards

**Affiliations:** QIMR Berghofer Medical Research Institute, Brisbane, Queensland, Australia

**Keywords:** allelic imbalance, GWAS, SNPs, transcription, breast cancer

Recent genome-wide associations studies (GWAS) have identified hundreds of common variants associated with the risk of developing breast cancer. However, a major challenge in the post-GWAS era is to understand the functional consequences of the identified SNPs. One of the main issues is that the majority of risk-associated SNPs fall in noncoding regions, and are predicted to function via *cis*-regulatory changes in gene expression [1]. A widely used approach to identify *cis*-acting regulatory SNPs (rSNPs) and their target gene(s) is expression quantitative trait loci (eQTL) mapping, where SNPs are tested for their association with mRNA levels. However, an alternative method is to compare the relative expression of the two alleles in individuals heterozygous for a transcribed SNP. Allelic imbalance (AI), or a deviation from the expected 1:1 ratio of alleles can offer increased sensitivity compared to standard eQTL, as the comparison is made within an individual, thereby minimising *trans*-acting environmental and genetic factors [2].

Accumulating evidence indicates that AI attributed to genotype variation is common in normal human tissue and is typically tissue-specific. High levels of AI have also been detected in the majority of cancer samples, likely arising from underlying DNA copy number alterations. In breast cancer, AI of *BRCA1*/*2* expression is associated with an increased risk of developing the disease [3]. AI at other loci has also been implicated in breast cancer prognosis and response to chemotherapy. A new paper in *Oncotarget* by Hamdi et al [4] has now identified 313 rSNPs showing evidence of association with AI selected from 175 genes involved in cancer etiology. Thirteen SNPs were associated with overall breast cancer risk and three reached *P*<10^-4^ significance after Bonferroni correction. Notably, the authors identified a novel breast cancer susceptibility locus at 4q21 (rs11099601), which has subsequently been confirmed in the most recent GWAS for breast cancer [5]. These results provide a good example of how AI can be used to identify new susceptibility loci and help pinpoint the individual causal regulatory variants and genes contributing to the disease association.

Functional annotation of the 4q21 locus using a combination of genetic, epigenomic and gene expression data derived from breast cells predicted several target genes, including *HELQ, FAM175A, MRPS18C* and *HPSE* [4]. *HELQ* and *FAM175A* encode proteins involved in pathways for DNA repair, making them plausible candidate breast cancer susceptibility genes. At present, there is little evidence that *MRPS18C* or *HPSE* are involved in tumorigenesis and will require additional functional studies to determine their role (if any) in disease. Of note, *FAM157A* was the only gene at the locus to show AI, but *cis*-eQTL analyses detected no significant associations. This discrepancy is likely due to the use of LCLs for the AI compared with breast-derived samples for the eQTL studies. It is well documented that *cis*-regulatory variants can effect gene expression in a cell type-specific manner [6]. An obvious future direction will be to perform the AI in normal breast tissue and breast cancer samples, which would provide further support for *FAM175A* as a breast cancer risk gene and/or may identify additional genes contributing to risk at this locus. Inconsistency between the AI and eQTL studies could also be due to different sample sizes and measurement of different isoforms depending on the microarray probe design.

The recent completion of the OncoArray [5], the largest breast cancer GWAS to date, together with expanding catalogs of rare and acquired variants from whole genome sequencing efforts, means the number of noncoding variants associated with breast cancer will rise. The plethora of high-throughput data generated through projects such as ENCODE [7] and Roadmap Epigenomics [8] will significantly accelerate the functional annotation of these variants. However, clearly additional datasets in more diverse breast cell types are needed to ensure that cell type-specific effects are captured. The next challenge will then be to identify the key genes whose expression is affected by these SNPs and to specifically test the allele-specific effect of SNPs on target transcript levels. This study [4] and many others highlight the need to apply multiple complementary approaches, in biologically relevant tissues, to identify the causal SNPs and genes driving GWAS associations. Ongoing efforts to integrate robust genetic data with information from diverse -omics initiatives will continue to shed light on the genes and mechanisms underlying the biology of complex traits.
